# Investigating the Mediating Role of Executive Function in the Relationship Between ADHD and DCD Symptoms and Depression in Adults

**DOI:** 10.1007/s10803-023-06148-7

**Published:** 2023-11-15

**Authors:** Maria C. Broletti, Christina Efthymiou, Aja Louise Murray, Emily McDougal, Sinéad M. Rhodes

**Affiliations:** 1 Department of Psychology, University of Edinburgh, Edinburgh, UK; 2 Child Life and Health/Centre for Clinical Brain Sciences, University of Edinburgh, Royal Hospital for Children and Young People, Edinburgh, EH16 4TS UK; 3 Evidence Based Practice Unit, Anna Freud and University College London, London, UK

**Keywords:** Attention deficit hyperactivity disorder, Developmental co-ordination disorder, Dyspraxia, Executive function, Depression

## Abstract

This study was designed to test the effects of Attention Deficit Hyperactivity Disorder (ADHD) and Developmental Co-ordination Disorder (DCD) on depression levels and investigate the mediating role of executive function (EF) in adults. Adults with ADHD, DCD, and ADHD + DCD (*N* = 139) completed self-report measures of ADHD, DCD, depression, and EF. There were distinct profiles of EF across diagnostic groups, and higher depression symptoms in adults with ADHD + DCD than DCD alone. All EF domains were predicted by ADHD symptoms, and several by DCD symptoms. ADHD and DCD symptoms, and most EF domains, predicted depression symptoms. Overall EF difficulties fully mediated the relationships between ADHD/DCD and depression symptoms. Several specific EF domains relating to behavioural regulation and metacognition also showed full/partial mediating effects. The mediating role of EF difficulties between these neurodevelopmental conditions and depression symptoms has implications for their understanding and treatment, suggesting that targeting EF may be important for preventing co-occurring depression.

**Supplementary Information:**

The online version contains supplementary material available at 10.1007/s10803-023-06148-7.

Attention Deficit Hyperactivity Disorder (ADHD) and Developmental Co-ordination Disorder (DCD; also known as Dyspraxia) are two distinct but related neurodevelopmental conditions. These conditions are typically diagnosed in childhood, with prevalence rates of about 1–2% each diagnosed in the UK (Lingam et al., 2009; Russell et al., 2014; Sayal et al., 2018). ADHD is characterised by pervasive difficulties with inattention and/or hyperactivity/impulsivity, which can impact academic, occupational and social outcomes. The core feature of DCD is difficulties with co-ordinated motor skills that affect daily activities relating to school/work, socialising, and physical health (American Psychiatric Association [APA], 2013). ADHD persists into adulthood for, on average, 43% of adults (Di Lorenzo et al., 2021), and DCD for around 30–70% of adults (Tal-Saban & Kirby, 2018). Some individuals may also not receive a diagnosis until adulthood due to late detection or presentation of symptoms (Cleaton et al., 2021; Magnin & Maurs, 2017; Taylor et al., 2022).

ADHD and DCD frequently co-occur, with estimated co-occurrence rates of up to 50% in each case (Blank et al., 2019; Goulardins et al., 2017). Additionally, an overlap can be seen between the core symptomatology as there have been reports of motor difficulties in ADHD (Goulardins et al., 2013; Langmaid et al., 2013; Papadopoulos et al., 2014) and of cognitive difficulties in DCD (Chen et al., 2009; Tal-Saban et al., 2014). However, only a few of these studies recognise the potential co-occurrence of an ADHD + DCD diagnosis and in those cases primarily do so to screen out such participants rather than investigate ADHD + DCD co-occurrence (Langmaid et al., 2013; Papadopoulos et al., 2014). When co-occurrence is not considered, there is a risk that findings seemingly relating to one condition are confounded by the presence of the other. This could mean that cognitive features that are attributed to one condition are in reality either related to another co-occurring issue (Goulardins et al., 2017) or, as is increasingly being recognised for executive functions, are better characterised as having transdiagnostic relevance (Astle et al., 2022). However, screening out those with co-occurring ADHD + DCD makes samples non-representative of ADHD and DCD populations and misses opportunities to illuminate the implications of their co-occurrence for important outcomes associated with each condition, such as depression.

ADHD and DCD are both characterised by difficulties in executive function (EF). EF is an umbrella term for a wide range of higher-order cognitive processes to perform tasks, achieve goals, socialise, and self-regulate emotions and behaviour (e.g., inhibition, working memory, planning; Goldstein et al., 2014). EF difficulties have been reported in research studies of adults diagnosed with ADHD (Barkley & Murphy, 2011), and there are positive associations between EF difficulties and ADHD symptoms (Silverstein et al., 2020). Similarly, adults with DCD have difficulties in EF compared to their neurotypical peers (Tal-Saban et al., 2012, 2014), and motor and EF difficulties are positively correlated (Rosenblum, 2013). However, only Tal-Saban et al. (2014) have considered the potential co-occurrence of attention and motor difficulties and controlled for such in analyses. Research investigating ADHD and DCD in isolation compared to groups with co-occurring ADHD + DCD provide some further insight into EF differences between these diagnoses. Rohr et al. (2023) reported that children with ADHD and ADHD + DCD have more challenges with the behavioural regulation aspects of EF than children with DCD. Conversely, Meachon et al. (2021) found no differences between adults with ADHD, DCD, and ADHD + DCD on a performance-based measure of inhibition. Further exploration of the differences between the EF profiles of ADHD, DCD, and ADHD + DCD in adults is thus needed.

Depression is a major mental health risk for adults with ADHD and/or DCD, with higher levels of depression-related markers reported in comparison to the neurotypical population (Hill & Brown, 2013; Landgren et al., 2021; Powell et al., 2021). ADHD and depression symptom severity are correlated (Powell et al., 2021), as are DCD symptoms and psychological distress (Li et al., 2019). An additive effect is observed in those with a dual diagnosis of ADHD + DCD, with parent reports (Missiuna et al., 2014) and twin studies (Piek et al., 2007) indicating that children with ADHD + DCD have greater levels of depression symptoms than those with ADHD or DCD alone. A comparison between adults with single and co-occurring ADHD and DCD has not yet been conducted.

EF is implicated as part of the cognitive difficulties observed in clinical depression (Rock et al., 2014). There are some suggestions of a predictive effect of EF difficulties on depression with regards to working memory (Letkiewicz et al., 2014) and shifting (Stange et al., 2017), although a bi-directional relationship has been found in a longitudinal study with older adults (Zainal & Newman, 2021). As an accompanying feature of ADHD and DCD, EF difficulties may be one pathway that increases the risk of depression in these conditions; research into understanding the relations between EF and depression is therefore warranted.

Previous research has shown that aspects of EF that individuals draw on in everyday situations predicts indices of depression in adults with ADHD. Difficulties with self-management of time has been shown to be associated with an increased risk of depression, while difficulties with self-organisation/problem-solving are also associated with increased depression symptoms (Knouse et al., 2013). Similarly, a study with university students found that organisation and strategic planning predicted mood symptoms, and that the association between ADHD and mood symptoms weakens once controlling for everyday functioning and EF (Mohamed et al., 2021). EF difficulties relating to behavioural regulation and metacognition also appear to play a mediating role in the relation between DCD and depression symptoms in children (Omer & Leonard, 2021), and have been implicated in the relationship between ADHD and quality of life in adults, whereby ADHD negatively affects quality of life through EF difficulties and then via depression symptoms (Zhang et al., 2021).

Given the commonalities between ADHD and DCD in diagnostic symptoms, and research suggesting a role of EF difficulties in depression, further research that examines them together is warranted. The current study had two primary aims: (a) to examine differences in EF profiles and depression levels between ADHD and DCD in relation to single and co-occurring diagnoses and symptomatology in adults; and (b) to examine the predictive effect of aspects of EF drawn on in everyday life on depression symptoms, and whether EF mediates the relationship between ADHD or DCD symptoms and depression. Based on the current literature, the following hypotheses were determined and pre-registered prior to data analysis on the Open Science Framework (https://doi.org/10.17605/OSF.IO/QMY2G):

1. There would be significant differences across the domains of EF between diagnostic groups.
2. Depression symptoms would be significantly higher in the ADHD + DCD group than the ADHD and DCD groups.
3. Levels of ADHD and DCD symptoms would significantly predict overall EF, and depression symptoms, within the full sample and each diagnostic group.
4. Overall EF would significantly predict depression symptoms, within the full sample and each diagnostic group.

The predictive effect of ADHD and DCD symptoms on the specific domains of EF, and the predictive effects of each EF domain on depression symptoms, would also be explored within the full sample. Significant findings from these exploratory analyses, and the H3 and H4 analyses, would be further explored by testing the indirect relationships between ADHD/DCD and depression symptoms through the different EF domains.

## Method

### Ethics

Favourable Ethical Opinion was received from the University of Edinburgh PPLS Research Ethics Committee. Before commencing the study, participants provided informed consent. The study involved questionnaire measures assessing ADHD, DCD, depression, and EF difficulties, however they were not diagnostic in nature and no information on individual scoring was given to participants.

### Participants

Participants were recruited by advertising the study on social media channels, including several charities such as the ADHD Foundation, the Scottish ADHD Coalition, and the Dyspraxia Foundation. Eligibility requirements involved being over the age of 18 and having a formal diagnosis of ADHD and/or DCD. Four participants were excluded from the analysis due to incomplete survey responses or unmet eligibility requirements. The final dataset consisted of 139 participants. The sample comprised 66 participants with DCD, 50 with ADHD, and 23 with ADHD + DCD. Ages ranged from 18 to 70 years, with a mean of 31.7 years (*SD* = 10.1). Further demographic characteristics are detailed in Table 1.

**Table 1:** Participant demographic information

Demographic characteristic	Full sample	ADHD group	DCD group	ADHD + DCD group
Age, *M* (*SD*)	31.7 (10.1)	37.6 (11)	27.8 (6.93)	30.1 (9.69)
Gender, *n* (%)				
Male	35 (25.1%)	11 (22%)	16 (24.2%)	8 (34.8%)
Female	104 (74.8%)	39 (78%)	50 (75.8%)	15 (65.2%)
Nationality, *n* (%)				
British	117 (84.2%)	37 (74%)	59 (89.4%)	21 (91.3%)
European	3 (2.2%)	–	2 (3%)	1 (4.3%)
North American	14 (10.1%)	10 (20%)	3 (4.5%)	1 (4.3%)
Other	4 (2.9%)	2 (4%)	2 (3%)	–
Not specified	1 (.7%)	1 (2%)	–	–
English as first language^a^, *n* (%)	133 (95.7%)	46 (92%)	64 (97%)	23 (100%)
Age of first diagnosis, *n* (%)				
Childhood (3–17)	44 (31.7%)	4 (8%)	31 (47%)	9 (39.1%)
Adulthood (18–70)	94 (67.6%)	46 (92%)	34 (51.5%)	14 (60.9%)
Not specified	1 (.7%)	–	1 (1.5%)	–
Other diagnoses, *n* (%)				
Dyslexia	32 (23%)	2 (4%)	18 (27.3%)	12 (52.2%)
Autism	9 (6.4%)	4 (8%)	2 (3%)	3 (13%)
Learning difficulties	15 (10.8%)	4 (8%)	5 (7.6%)	6 (26.1%)
Depression	8 (5.8%)	7 (14%)	1 (1.5%)	–
Anxiety	6 (4.3%)	5 (10%)	1 (1.5%)	–
Posttraumatic stress disorder	3 (2.2%)	3 (6%)	–	–
Borderline personality disorder	2 (1.4%)	2 (4%)	–	–
Bulimia	1 (.7%)	–	1 (1.5%)	–

*N* = 139 (*n* = 50 for ADHD, *n* = 66 for DCD, and *n* = 23 for ADHD + DCD groups)

^a^Reflects number/percentage of participants who answered “yes”

### Materials and Procedure

The study was administered online via the survey platform Qualtrics, accessed through a link and password emailed to participants. Following the information and consent page, participants completed the study measures as outlined below.

#### Demographic Questions

Participants were asked about their age; gender; nationality; if English was their first language; neurodevelopmental diagnoses including DCD, ADHD, Dyslexia, Autism, and Learning Difficulties; other diagnoses; type of clinician that diagnosed them for each diagnosis; and what year(s) they were diagnosed. Information about diagnosis was used to determine the diagnostic group that participants were assigned to.

#### ADHD Symptoms

The Adult ADHD Self-Report Scale (ASRS; Kessler et al., 2005) is an 18-item self-report scale for adult ADHD that assesses inattention and hyperactivity-impulsivity over the prior six months. Participants rated how often they experienced these symptoms on a 5-point scale (0 = *Never*, 1 = *Rarely*, 2 = *Sometimes*, 3 = *Often*, 4 = *Very often*). Cronbach’s alpha indicated excellent internal consistency in the current sample (α = .90). For descriptive statistics regarding clinical cut-off points, the ASRS Screener (Kessler et al., 2005) was used which scores the first six items of the ASRS. The 0–5 scoring system is replaced with a 0–1 system, with scores ≥ 4 indicating possible ADHD.

#### DCD Symptoms

The Adult Developmental Co-ordination Disorder/Dyspraxia Checklist (ADC; Kirby et al., 2010) is a 40-item self-report screening questionnaire for DCD. It asks about diagnostic-related difficulties as a child (Subscale A; 10 items) and an adult (Subscales B and C; 20 items). Participants rated how often these difficulties occurred for them on a 4-point scale (0 = *Never*, 1 = *Sometimes*, 2 = *Frequently*, 3 = *Always*). As two items referred to driving (Items 25 and 32), a ‘Not driving/applicable’ option was included, with the first of the two items allowing participants to provide their own reasoning as to why. Meachon et al.’s (2022) method was adapted, whereby a prorated score was given to those who selected these responses for both items based on their reasoning (e.g., financial reasons receiving a lower score, co-ordination difficulties receiving a higher score). For clinical cut-offs, a score ≥ 17 on Subscale A and a total score ≥ 56 suggest clinical difficulties and probable DCD (Kirby et al., 2013; Purcell et al., 2015). To create a measure for *current* DCD symptoms, only items from Subscales B and C were included. Cronbach’s alpha indicated good internal consistency for this combined measure (α = .85) and excellent internal consistency (α = .90) for the full scale in the current sample.

#### Depression Symptoms

The Mood and Feelings Questionnaire (MFQ; Costello & Angold, 1988) is a 33-item self-report questionnaire that assesses depression symptoms over the prior 2 weeks. Participants rated how true each statement was on a 3-point scale (0 = *Not true*, 1 = *Sometimes*, 2 = *True*). Cronbach’s alpha indicated excellent internal consistency (α = .94) in the current sample. For clinical cut-offs, a score ≥ 33 signifies depression.

#### Executive Function

The Behavior Rating Inventory of Executive Function-Adult Version (BRIEF-A; Roth et al., 2005) is a standardised measure of adult’s EF in their everyday environment. The self-report form was used in this study. The Global Executive Composite (GEC) refers to the full 75-item scale and was used to assess everyday EF. Individual domains of EF were assessed through two index scales and their respective subscales: the Behavioural Regulation Index (BRI; subscales include Inhibit, Shift, Emotional Control, Self-Monitor) and the Metacognition Index (MI; subscales include Initiate, Working Memory, Plan/Organize, Task Monitor, Organization of Materials). Participants rated how often they experienced difficulties with these everyday EFs over the previous month (1 = *Never*, 2 = *Sometimes*, 3 = *Often*). Cronbach’s alpha indicated excellent internal consistency (α = .95) in the current sample. As there are separate BRIEF-A norms for different age groups, raw scores were transformed into T-scores according to the professional manual (Roth et al., 2005). A GEC score ≥ 65 marks the clinical cut-off point.

### Statistical Analysis

Analysis plans were preregistered on the Open Science Framework (https://doi.org/10.17605/OSF.IO/QMY2G). Statistical analysis was conducted using R, including its *stats* package (version 4.0.2; R Core Team, 2020), as well as the *lavaan* package (version 0.6.11; Rosseel, 2012). To test differences between diagnostic groups, one-way Analyses of Covariance (ANCOVAs) were conducted for each domain of everyday EF and depression symptoms. Significant findings were followed up with pairwise comparisons using Tukey’s HSD, which adjusted for multiple comparisons. ADHD and DCD symptoms as predictors of the different domains of EF and depression symptoms were examined using multiple linear regressions. Simple linear regressions were conducted to test each domain of EF as a predictor of depression symptoms. Age, age of diagnosis (dummy coded as: 0 = childhood; 1 = adulthood), and gender (dummy coded as: 0 = male, 1 = female) were included as covariates in all analyses (see Supplementary Material for results without covariates). Where there were significant relationships between variables within the regression analyses, path mediation analysis was used to test the indirect relationship between ADHD/DCD symptoms and depression symptoms through EF (see Fig. S1 in Supplementary Material). To test the statistical significance of the indirect effects, 95% bootstrapped confidence intervals were used, based on 1,000 draws.

Missing responses on the BRIEF-A were handled using the guidelines of the professional manual (Roth et al., 2005), while those on the remaining questionnaire measures were dealt with using mean imputation. Most analyses met assumptions. There was an influential outlier (Cook’s distance > .5) in the simple regression predicting depression symptoms by everyday EF within the ADHD + DCD group. The outlier was removed from the statistical model as it changed significance in findings. Linearity was also partly violated for this model, as well as for the multiple regressions predicting depression symptoms by ADHD and DCD symptoms in the DCD and ADHD + DCD groups. As such, these models were run using structural equation modelling (SEM) and a robust maximum likelihood estimator instead. The assumption of homoscedasticity was violated for the multiple regression predicting metacognition by ADHD and DCD symptoms, and so was conducted using SEM and a weighted least squares estimator.

## Results

### Descriptive Statistics

Descriptive statistics of study measures pertaining to ADHD, DCD, and depression symptoms, and everyday EF are presented in Table 2, as are the number of participants who met cut-off points for ADHD, DCD, depression, and EF difficulties.

**Table 2:** Summary of ASRS, ADC, MFQ, and GEC scores with clinical cut-off points

Measure	Full sample	ADHD group	DCD group	ADHD + DCD group
ASRS				
* M* (*SD*)	49.6 (11.5)	55.6 (8.58)	43.5 (11.1)	54.1 (9.31)
Clinical cut-off, *n* (%)	106 (76%)	47 (94%)	38 (57.6%)	21 (91.3%)
ADC				
* M* (*SD*)	53 (12.7)	50.1 (13)	52.9 (11.5)	59.9 (13.3)
Clinical cut-off, *n* (%)	118 (84.9%)	36 (72%)	61 (92.4%)	21 (91.3%)
MFQ				
* M* (*SD*)	28.8 (14.4)	28.3 (13.6)	26.4 (13.3)	37.1 (16.6)
Clinical cut-off, *n* (%)	75 (54%)	29 (58%)	29 (43.9%)	17 (73.9%)
GEC				
* M* (*SD*)	76.9 (11.1)	81.9 (8.76)	71.2 (10.8)	82.2 (8.46)
Clinical cut-off, *n* (%)	123 (88.5%)	49 (98%)	51 (77.3%)	23 (100%)

*N* = 139 (*n* = 50 for ADHD, *n* = 66 for DCD, and *n* = 23 for ADHD + DCD groups)

### Diagnostic Differences in EF and Depression Symptoms

The ANCOVAs indicated significant effects of diagnosis on each domain of everyday EF (*p* < .05) and depression symptoms (*p* = .009; see Table 3). Of the covariates, age had a significant effect on metacognition (*F*(1,132) = 6.57, *p* = .01, η_p_^2^ = .05) and planning/organising (*F*(1,132) = 6.96, *p* = .009, η_p_^2^ = .05), as did gender on working memory (*F*(1,132) = 3.98, *p* = .048, η_p_^2^ = .03). Pairwise comparisons revealed that for shifting and emotional control, the ADHD + DCD group had significantly greater difficulties than the DCD group (*p* = .005), however there were no differences between the ADHD and either ADHD + DCD or DCD groups (*p* > .05) (see Table 4). There were no significant differences between any of the groups on organisation of materials (*p* > .05). Of the remaining domains of EF, there were no significant differences between the ADHD and ADHD + DCD groups (*p* > .05), but both groups had significantly greater difficulties than the DCD group (*p* < .05). With regards to depression symptoms, the ADHD + DCD group had significantly higher levels than the DCD group (*p* = .005), however there were no significant differences between the ADHD and either ADHD + DCD or DCD groups (*p* > .05).

**Table 3:** Adjusted means, standard errors, and ANCOVAs of group differences in EF (BRIEF-A) and Depression Symptoms (MFQ)

Measure	ADHD group		DCD group		ADHD + DCD group		*F*	*p*	η_p_^2^
	*M*	*SE*	*M*	*SE*	*M*	*SE*			
BRIEF-A									
GEC	80.7	1.72	71.4	1.35	82.2	2.08	21.53	< .001	.25
BRI	76.2	1.89	66.5	1.49	77.4	2.28	19.42	< .001	.23
MI	79.9	1.77	72.7	1.39	81.9	2.14	15.42	< .001	.19
Inhibit	73.7	1.78	62.0	1.40	73.8	2.15	26.5	< .001	.29
Shift	73.6	2.05	67.7	1.62	76.9	2.48	7.65	< .001	.10
EC	70.2	1.97	64.7	1.56	71.2	2.39	7.65	< .001	.10
S-M	69.6	2.17	59.1	1.71	68.6	2.63	11.66	< .001	.15
Initiate	75.1	1.88	66.3	1.48	75.8	2.27	13.56	< .001	.17
WM	82.5	1.74	77.3	1.37	84.8	2.11	7.36	< .001	.10
P/O	77.9	1.82	69.7	1.44	78.9	2.20	15.89	< .001	.19
TM	75.8	1.85	68.4	1.46	76.6	2.24	14.34	< .001	.18
OM	67.6	2	65.6	1.58	70.9	2.42	3.13	.047	.05
MFQ	28.9	2.48	25.7	1.96	36.8	3	4.93	.009	.07

Means and standard errors are adjusted for age, age of diagnosis, and gender

*EC* emotional control, *S-M* self-monitor, *WM* working memory, *P/O* plan/organize, *TM* task monitor, *OM* organization of materials

**Table 4:** Pairwise comparisons of EF (BRIEF-A) and Depression Symptoms (MFQ) between Diagnostic Groups

Measure	Group	Mean difference	*p*
BRIEF-A			
GEC	ADHD – DCD	9.28	< .001
	ADHD + DCD – DCD	10.78	< .001
	ADHD + DCD – ADHD	1.5	.83
BRI	ADHD – DCD	9.77	< .001
	ADHD + DCD – DCD	10.94	< .001
	ADHD + DCD – ADHD	1.17	.91
MI	ADHD – DCD	7.16	.004
	ADHD + DCD – DCD	9.19	< .001
	ADHD + DCD – ADHD	2.03	.73
Inhibit	ADHD – DCD	11.76	< .001
	ADHD + DCD – DCD	11.85	< .001
	ADHD + DCD – ADHD	.09	.99
Shift	ADHD – DCD	5.92	.06
	ADHD + DCD – DCD	9.24	.004
	ADHD + DCD – ADHD	3.32	.54
EC	ADHD – DCD	5.54	.07
	ADHD + DCD – DCD	6.55	.049
	ADHD + DCD – ADHD	1.01	.94
S-M	ADHD – DCD	10.53	< .001
	ADHD + DCD – DCD	9.46	.006
	ADHD + DCD – ADHD	1.08	.94
Initiate	ADHD – DCD	8.88	< .001
	ADHD + DCD – DCD	9.5	.001
	ADHD + DCD – ADHD	.63	.97
WM	ADHD – DCD	5.23	.046
	ADHD + DCD – DCD	7.46	.008
	ADHD + DCD – ADHD	2.23	.68
P/O	ADHD – DCD	8.2	.001
	ADHD + DCD – DCD	9.2	.001
	ADHD + DCD – ADHD	1	.93
TM	ADHD – DCD	7.47	.004
	ADHD + DCD – DCD	8.19	.005
	ADHD + DCD – ADHD	.72	.96
OM	ADHD – DCD	2.08	.68
	ADHD + DCD – DCD	5.32	.14
	ADHD + DCD – ADHD	3.24	.54
MFQ	ADHD – DCD	3.16	.56
	ADHD + DCD – DCD	11.08	.005
	ADHD + DCD – ADHD	7.92	.09

*EC* emotional control, *S-M* self-monitor, *WM* working memory, *P/O* plan/organize, *TM* task monitor, *OM* organization of materials

### EF and Depression Symptoms as Predicted by ADHD and DCD Symptoms

The multiple linear regression models testing the predictive effects of ADHD and DCD symptoms on each domain of EF and depression symptoms were all significant (*p* < .05). ADHD symptoms was entered first into models, followed by DCD symptoms, then control variables. Where DCD symptoms were a significant predictor, incremental F-ratio tests confirmed they explained a significant amount of variance (*p* < .05) over and above ADHD symptoms. Both ADHD and DCD symptoms were significant predictors of EF and depression symptoms in the full sample (*p* < .01), as were the covariates age (β = .13, *p* = .03) and gender (β = − .14, *p* = .009) for everyday EF. The regressions conducted within each diagnostic group revealed that ADHD symptoms was a significant predictor of EF in the DCD group (*p* < .001), while DCD symptoms was a significant predictor of EF in the ADHD and ADHD + DCD groups (*p* < .05) and of depression symptoms in the ADHD group (*p* = .01; see Table 5).

**Table 5:** Prediction of overall EF (GEC) and Depression Symptoms (MFQ) from ADHD Symptoms (ASRS) and DCD Symptoms (ADC)

Predictor	GEC			MFQ		
	Estimate (β)	95% CI	*p*	Estimate (β)	95% CI	*p*
	Full sample					
ASRS	.65	[.51, .75]	< .001	.26	[.09, .57]	.008
ADC	.21	[.07, .29]	.001	.27	[.09, .52]	.005
	ADHD group					
ASRS	.18	[− .1, .46]	.2	− .11	[− .75, .41]	.56
ADC	.62	[.24, .6]	< .001	.47	[.12, .88]	.01
	DCD group					
ASRS	.71	[.5, .88]	< .001	.36	[− .05, .91]	.08
ADC	.12	[− .07, .3]	.23	.17	[− .12, .5]	.22
	ADHD + DCD group					
ASRS	.41	[.00, .75]	.05	.24	[− .49, 1.33]	.37
ADC	.45	[.03, .54]	.03	.19	[− .38, .85]	.45

Analyses were controlled for age, age of diagnosis, and gender

β Standardized coefficient

Multiple linear regression model exploration of the individual domains of EF, including covariates, revealed that ADHD symptoms was a significant predictor of each domain of EF (*p* < .05), and DCD symptoms for metacognition, shifting, working memory, planning/organising, task monitoring, and organisation of materials (*p* < .05; see Table 6). Age was also a significant predictor of metacognition (β = .24, *p* < .001), shifting (β = .16, *p* = .04), working memory (β = .15, *p* = .02), and planning/organising (β = .28, *p* < .001), and gender for behavioural regulation (β =  − .16, *p* = .009), inhibition (β = − .22, *p* < .001), shifting (β = − .16, *p* = .03), self-monitoring (β = − .15, *p* = .04), and planning/organising (β = − .14, *p* = .03).

**Table 6:** Prediction of EF (BRIEF-A) from ADHD Symptoms (ASRS) and DCD Symptoms (ADC)

Predictor	Estimate (β), 95% CI, *p*										
	BRI	MI	Inhibit	Shift	EC	S-M	Initiate	WM	P/O	TM	OM
ASRS	β = .64, [.52, .82], *p* < .001	β = .55, [.37, .69], *p* < .001	β = .83, [.72, .98], *p* < .001	β = .27, [.11, .47], *p* = .002	β = .42, [.24, .62], *p* < .001	β = .58, [.47, .85], *p* < .001	β = .58, [.41, .75], *p* < .001	β = .58, [.41, .75], *p* < .001	β = .48, [.33, .64], *p* < .001	β = .5, [.35, .65], *p* < .001	β = .2, [.01, .39], *p* = .04
ADC	β = .11, [− .03, .24], *p* = .12	β = .25, [.11, .41], *p* < .001	β = − .06, [− .17, .05], *p* = .31	β = .38, [.2, .53], *p* < .001	β = .04, [− .13, .21], *p* = .66	β = .08, [− .09, .25] *p* = .34	β = .07, [− .08, .21], *p* = .4	β = .18, [.03, .27], *p* = .02	β = .24, [.09, .36], *p* = .002	β = .25, [.09, .36], *p* = .001	β = .29, [.1, .43], *p* = .002

Analyses were controlled for age, age of diagnosis, and gender

β Standardized coefficient, *EC* emotional control, *S-M* self-monitor, *WM* working memory, *P/O* plan/organize, *TM* task monitor, *OM* organization of materials

### Depression Symptoms as Predicted by EF

The simple linear regression models examining each domain of everyday EF as a predictor of depression symptoms were all significant (*p* < .01), except for the model with organisation of materials as the predictor (*p* > .05). The regressions conducted within each diagnostic group suggested that EF was a significant predictor of depression symptoms for adults diagnosed with ADHD-only and DCD-only (*p* < .001), however EF did not significantly predict depression symptoms for adults diagnosed with ADHD + DCD (*p* > .05). The remaining domains of everyday EF significantly predicted depression symptoms in the full sample (*p* < .001), except for organisation of materials (*p* > .05; see Table 7). Age was also a significant predictor in models with behavioural regulation (β = − .18, *p* = .02), metacognition (β = − .22, *p* = .01), initiation (β = − .18, *p* = .03), and planning/organising (β = − .24, *p* = .007) as the EF predictor.

**Table 7:** Prediction of Depression Symptoms (MFQ) from EF (BRIEF-A)

Predictor	MFQ		
	Estimate (β)	95% CI	*p*
	Full sample		
GEC	.55	[.52, .91]	< .001
BRI	.58	[.52, .87]	< .001
MI	.45	[.37, .79]	< .001
Inhibit	.43	[.32, .72]	< .001
Shift	.44	[.34, .71]	< .001
EC	.56	[.5, .86]	< .001
S-M	.36	[.22, .57]	< .001
Initiate	.5	[.44, .82]	< .001
WM	.34	[.23, .69]	< .001
P/O	.47	[.38, .78]	< .001
TM	.37	[.25, .67]	< .001
OM	.14	[− .05, .39]	.12
	ADHD group		
GEC	.58	[.51, 1.29]	< .001
	DCD group		
GEC	.47	[.29, .86]	< .001
	ADHD + DCD group		
GEC	.27	[− .18, 1.34]	.13

Analyses were controlled for age, age of diagnosis, and gender

β Standardized coefficient, *EC* emotional control, *S-M* self-monitor, *WM* working memory, *P/O* plan/organize, *TM* task monitor, *OM* organization of materials

### Mediation Effects of EF in the Relation Between ADHD/DCD and Depression Symptoms

Following up on the significant findings from the results above, simple mediation analyses were conducted to test the indirect relationships between (1) ADHD and depression symptoms via each domain of EF, excluding organisation of materials; and (2) DCD and depression symptoms via each of the following: overall EF, metacognition, shifting, working memory, planning/organising, and task monitoring.

As shown in Table 8, overall EF, behavioural regulation, metacognition, shifting, self-monitoring, emotional control, initiation, and planning/organising respectively acted as significant mediators of associations between ADHD and depression symptoms, however inhibition, working memory, and task monitoring did not show mediating effects. Overall EF, behavioural regulation, metacognition, emotional control, initiation, and planning/organising displayed full mediating effects, while shifting and self-monitoring showed partial mediating effects. Additionally, overall EF showed significant full mediating effects between DCD and depression symptoms, while metacognition, shifting, and planning/organising respectively showed significant partial mediating effects. Working memory and task monitoring did not present mediating effects.

**Table 8:** Results of the Simple Mediation Models (ADHD/DCD Symptoms → EF → Depression Symptoms)

Mediation model	Estimate (β), 95% CI				
	Effect of IV on M	Effect of M on DV	Total Effect	Direct Effect	Indirect Effect
	ADHD symptoms → EF → Depression symptoms				
ASRS → GEC → MFQ	β = .71, [.51, .71]*	β = .38, [.1, 1.09]*	β = .41, [.25, .79]*	β = .14, [− .28, .59]	β = .27, [.06, .7]*
ASRS → BRI → MFQ	β = .7, [.6, .85]*	β = .58, [.42, .96]*	β = .41 [.23, .77]*	β = .01, [− .33, .32]	β = .4, [.28, .74]*
ASRS → MI → MFQ	β = .68, [.52, .77]*	β = .32, [.04, .73]*	β = .41, [.27, .82]*	β = .19, [− .04, .6]	β = .22, [.03, .47]*
ASRS → Inhibit → MFQ	β = .79, [.72, .92]*	β = .28, [− .01, .68]	β = .41, [.24, .8]*	β = .19, [− .14, .62]	β = .22, [− .01, .57]
ASRS → Shift → MFQ	β = .48, [.35, .67]*	β = .33, [.18, .58]*	β = .41, [.26, .79]*	β = .25, [.06, .6]*	β = .16, [.09, .33]*
ASRS → EC → MFQ	β = .45, [.27, .62]*	β = .47, [.38, .77]*	β = .41, [.24, .77]*	β = .2, [− .02, .53]	β = .21, [.14, .4]*
ASRS → S-M → MFQ	β = .62, [.57, .85]*	β = .2, [.01, .41]*	β = .41, [.26, .81]*	β = .29, [.07, .69]*	β = .12, [.004, .31]*
ASRS → Initiate → MFQ	β = .62, [.46, .76]*	β = .41, [.27, .73]*	β = .41, [.25, .79]*	β = .16, [− .08, .51]	β = .25, [.17, .47]*
ASRS → WM → MFQ	β = .68, [.47, .74]*	β = .13, [− .11, .5]	β = .41, [.24, .79]*	β = .33, [.11, .72]*	β = .09, [− .07, .28]
ASRS → P/O → MFQ	β = .62, [.49, .75]*	β = .34, [.15, .7]*	β = .41, [.24, .78]*	β = .2, [− .07, .59]	β = .21, [.1, .45]*
ASRS → TM → MFQ	β = .63, [.5, .76]*	β = .18, [− .06, .52]	β = .41, [.24, .78]*	β = .3, [.07, .68]*	β = .11, [− .04, .33]
	DCD symptoms → EF → Depression symptoms				
ADC → GEC → MFQ	β = .5, [.25, .51]*	β = .36, [.2, .94]*	β = .4, [.28, .63]*	β = .23, [− .05, .5]	β = .18, [.06, .44]*
ADC → MI → MFQ	β = .53, [.36, .58]*	β = .32, [.08, .77]*	β = .4, [.26, .63]*	β = .24, [.04, .48]*	β = .17, [.04, .35]*
ADC → Shift → MFQ	β = .52, [.37, .63]*	β = .32, [.16, .61]*	β = .4, [.27, .63]*	β = .24, [.03, .47]*	β = .17, [.27, .63]*
ADC → WM → MFQ	β = .48, [.28, .53]*	β = .18, [− .04, .57]	β = .4, [.27, .62]*	β = .32, [.15, .56]*	β = .09, [− .01, .23]
ADC → P/O → MFQ	β = .49, [.34, .58]*	β = .34, [.15, .7]*	β = .4, [.28, .64]*	β = .24, [.05, .47]*	β = .17, [.07, .33]*
ADC → TM → MFQ	β = .5, [.35, .57]*	β = .21, [− .003, .55]	β = .4, [.27, .63]*	β = .3, [.12, .54]*	β = .11, [− .001, .25]

Analyses were controlled for age, age of diagnosis, and gender

β Standardized coefficient, *IV* independent variable, *M* mediator, *DV* dependent variable, *EC* emotional control, *S-M* self-monitor, *WM* working memory, *P/O* plan/organize, *TM* task monitor

*Significant confidence interval

## Discussion

The present study investigated EF and depression symptoms, and the relationship between them, in single and co-occurring ADHD and DCD in adults. Diagnostic and symptomatology patterns in EF revealed similar but distinct cognitive profiles amongst ADHD, DCD, and ADHD + DCD in adults. Levels of ADHD symptoms predicted difficulties in all domains of EF, and those with a diagnosis of ADHD mostly had greater difficulties than those without. The predictive effect of ADHD symptoms on EF difficulties corresponds with previous findings in a clinical sample of adults with ADHD (Leikauf & Solanto, 2017). The current study provided novel outcomes in determining which domains of everyday EF are associated with DCD symptoms in adults, as research has been limited to the association between everyday EF and DCD status in children and has focused on diagnosis rather than symptoms (Omer & Leonard, 2021). DCD symptoms mainly affected different aspects of metacognition (i.e., working memory, planning/organising, task monitoring, organising materials) which is in line with the type of EF difficulties reported in adult DCD (Tal-Saban & Kirby, 2018).

The diagnostic differences in EF profiles partly resembled findings in children with respect to behavioural regulation and inhibition, with the ADHD and ADHD + DCD groups showing greater difficulties than the DCD group (Rohr et al., 2023). Children also show these differences in difficulties with shifting and emotional control (Rohr et al., 2023), however the current study found that while the ADHD + DCD group had greater difficulties than the DCD group for shifting and emotional control, difficulty levels for the ADHD group were intermediate between these two groups. As the everyday EF challenges adults face are likely to be different from those experienced by children, it is important to examine how these relations between EF and ADHD/DCD manifest in adults. Interestingly, there were also no indications of additive effects reflected in the EF profiles. Domains of EF, such as working memory or planning/organising, were predicted by both ADHD and DCD symptomatology, but it was the adults with ADHD-only and ADHD + DCD who had greater difficulties than adults with DCD-only. Overall, the presentation of two diagnoses does not produce an additive effect on the cognitive profile of ADHD + DCD. In particular, the results suggest that the presence of ADHD is a key factor in EF difficulties in these groups and underline the importance of measuring ADHD symptoms in studies of DCD and EF, given that their co-occurrence may act as a confounding factor.

This study was the first to examine diagnostic differences with regards to depression in single and co-occurring ADHD and DCD in adults. Previous studies with children have reported that those with ADHD + DCD have higher depression levels than those with ADHD- or DCD-only (Missiuna et al., 2014; Piek et al., 2007), however, the current findings discovered something different. While the ADHD + DCD group had higher levels of depression symptoms than the DCD group, the levels in the ADHD group were intermediate between the ADHD + DCD and DCD groups with no significant differences between them. Thus, both ADHD and DCD symptoms contributed to the variance in depression symptoms, consistent with prior findings of ADHD symptoms as a predictor of depression symptoms in adults (Mohamed et al., 2021).

Contrary to what was hypothesised, none of the individual diagnostic groups showed an effect of both ADHD symptoms and DCD symptoms on EF and depression symptoms. Rather, only ADHD symptoms predicted EF difficulties in the DCD group; and only DCD symptoms predicted EF difficulties in the ADHD + DCD and ADHD groups, and depression symptoms in the ADHD group. Findings in the ADHD and DCD groups could be attributed to elevated symptoms: 57.6% of those in the DCD group met clinical cut-off points for ADHD, as did 72% of those in the ADHD group for DCD. Regardless of whether these participants indeed have undiagnosed ADHD/DCD, they may be missing out on relevant coping tools or support for ADHD/DCD symptoms that only come with a formal diagnosis. Adults with late-diagnosed ADHD have reported the positive impact of their newfound diagnosis in improving daily functioning and wellbeing as they can understand themselves better and seek support (Halleröd et al., 2015). However, the findings are difficult to interpret, considering the smaller sample sizes of each diagnostic group in comparison to the full sample and the fact that variation in EF and depression may be more constrained within groups that show elevated levels of each, resulting in range restriction effects. This particularly relates to the ADHD + DCD group (*n* = 23) where EF did not predict depression symptoms and 100% of the sample had elevated EF difficulties while 73.9% had elevated depression symptoms. Research with larger samples may provide more concrete conclusions.

In the full sample, depression symptoms were predicted by difficulties with all aspects of EF, apart from organisation of materials. This BRIEF-A subscale measures behaviours such as misplacing objects or not having belongings readily available for use, reflecting difficulties with keeping order in one’s surroundings necessary to function efficiently at home or work (Roth et al., 2005). Zhang et al. (2021) found no correlation between this aspect of EF and depression symptoms within a sample of adults with ADHD. While the cognitive aspects of organisation (as seen in planning/organising) may contribute to depression symptoms, it appears its environmental manifestation within organisation of materials does not.

Mediation analyses provided further insight into the effects of EF on depression symptoms. A major finding was the fully mediating effect of EF difficulties on the association between both symptoms of ADHD and depression, and symptoms of DCD and depression. This effect is thus imperative to understanding and supporting wellbeing in adults with ADHD and/or DCD, especially considering its connections to quality of life in ADHD (Zhang et al., 2021). Of the individual domains of EF, behavioural regulation fully mediated the relationship between ADHD and depression symptoms, as did emotional control. Difficulties with emotional lability (i.e., sudden, marked, inappropriate shifts in emotions) have also been found to partially mediate ADHD symptoms and depression symptoms in community ascertained adult samples (Murray et al., 2021). In the current study, self-monitoring and shifting acted as partial mediators between ADHD and depression symptoms, as did shifting for DCD and depression symptoms. The factors that may account for these mediational effects could include poor self-monitoring of social behaviours, which have been shown to mediate ADHD and depression across development in children (Feldman et al., 2017). Shifting could also be linked with emotional control (Roth et al., 2005) with negative reactions elicited by an inability to adapt to situations dependent on the ability to regulate emotions.

Metacognition and the related functions of initiation and planning/organising showed fully mediating effects on the relationship between ADHD and depression symptoms. These findings help extend existing research concerning similar metacognitive processes, such as the predictive effects of organisation and strategic planning on depression symptoms (Mohamed et al., 2021) and of self-management to time on the risk of depression (Knouse et al., 2013). Metacognition and planning/organising also partially mediated DCD and depression symptoms. The unknown mechanisms of these mediations could be due to the psychosocial implications of motor difficulties cited to play a role in developing depression in DCD (Cairney et al., 2013; Li et al., 2019). Metacognition may be implicated in academic/occupational functioning (e.g., planning/organising difficulties in procrastination; Rabin et al., 2011). Negative outcomes of difficulties with education/work have relevance for depression, such as higher levels of depression symptoms in unemployed compared to employed adults with DCD (Kirby et al., 2013). Noticeably, the results of the mediation analyses conflict with those in previous studies of children with DCD where behavioural regulation rather than metacognition has been shown to mediate relations between a DCD diagnosis and depression symptoms (Omer & Leonard, 2021). Thus, there may be a potential developmental shift in the impact of EF difficulties; however, this will need to be investigated in future research.

Mediating effects were absent for working memory and task monitoring in the relations between ADHD/DCD and depression symptoms, while inhibition did not display a mediating effect between ADHD and depression symptoms. While previous studies have pointed towards both the BRIEF-A indexes of behavioural regulation and metacognition as mediators in these relationships (Omer & Leonard, 2021; Zhang et al., 2021), their subscales had previously been unexplored until this study. Further research could test whether current findings can be replicated in different samples and, if so, why these particular EF domains do not produce a mediational effect.

### Limitations

There were some limitations to the study. First, as participants self-disclosed formal diagnoses of ADHD and/or DCD, there was no clinical confirmation of diagnoses. While the diagnostic thresholds of the ASRS and ADC could have been used to validate diagnoses, this method was not employed as it could exclude adults whose symptoms no longer meet clinical thresholds due to the help of medication or intervention. Second, a neurotypical comparison group was not included as the focus of the study was on differences between ADHD and DCD and the relationship between EF and depression within these single and co-occurring conditions rather than characterising symptom atypicality. Third, there was a disproportionate number of female participants compared to males (3:1), making the sample less representative in terms of gender; as well as an unequal distribution of other diagnoses amongst the ADHD, DCD, and ADHD + DCD groupings which may have unknowingly influenced results relating to group differences. Fourth, the assessment of EF using a self-rating scale rather than objective neuropsychological performance tests could be considered a limitation, however the current study was focused on aspects of EF individuals draw on in everyday real life situations. EF performance tests lack ecological validity due to their time-limited nature and attempts to isolate specific EFs which cannot account for the multi-faceted use of EF that is continuously influenced by daily life (Barkley & Murphy, 2010, 2011). In contrast, rating scales are more suitable as they rely on individuals’ own expertise of how they use EF in daily scenarios (Roth et al., 2005). Lastly, the use of a cross-sectional design limits the ability to ascertain causal effects of ADHD or DCD symptoms and EF on depression symptoms in this study. Nonetheless, these cross-sectional findings of mediational effects set the foundations for a more in-depth investigation, such as the collection of longitudinal data or application of causal mediation analysis.

### Implications and Future Research

The outcomes of this study highlight the need for further research on interventions that address difficulties with EF encountered by adults with ADHD and/or DCD, as a protective factor for mental health difficulties. Stimulant medication for ADHD symptoms could be useful, but have only been shown to improve EF to a certain extent and as such call for the use of adjacent psychosocial interventions (DuPaul et al., 2012). A transdiagnostic approach could be used in interventions, tailoring them towards an individual’s strengths and difficulties given the heterogeneity and common co-occurrence of neurodevelopmental conditions (Astle et al., 2022; Dalgleish et al., 2020). Improving understanding of EF difficulties and their role in increasing the risk of depression dependent on ADHD or DCD symptoms may be useful in reducing mental health difficulties for people with these conditions.

## Conclusions

This study found both similar but also differing effects of diagnosis/symptomatology of ADHD and DCD on EF and depression symptoms in adults. EF difficulties were found to predict levels of depression symptoms, showing full mediating effects on the relationship between ADHD/DCD symptoms and depression symptoms. The study also produced novel findings in identifying which aspects of EF used in everyday life produce these mediating effects. The findings have implications for moving forward with further transdiagnostic research and interventions concerned with reducing the risk of depression in adults with ADHD and DCD through the lens of common EF difficulties.

## Supplementary Information

Below is the link to the electronic supplementary material.

Supplementary file1 (DOCX 54 KB)
