# Supplementary material for: Investigating the Mediating Role of Executive Function in the Relationship Between ADHD and DCD Symptoms and Depression in Adults
Source: J Autism Dev Disord. 2023 Nov 15;54(12):4684–96. doi: 10.1007/s10803-023-06148-7 (PMC11549189; doi:10.1007/s10803-023-06148-7)
Supplement: Supplementary file 1 — Supplementary file1 (DOCX 54 KB) [file 10803_2023_6148_MOESM1_ESM.docx]

# **Investigating the Mediating Role of Executive Function in the Relationship between ADHD and DCD Symptoms and Depression in Adults**

Journal of Autism and Developmental Disorders

## Supplementary Figures

**Figure S1**

*Sample Mediation Models of Indirect Associations between ADHD/DCD and Depression Symptoms via EF*

DCD

symptoms

EF

Depression symptoms

ADHD

symptoms

EF

Depression symptoms

## Supplementary Results

## Diagnostic Differences in EF and Depression Symptoms

Without covariates, the individual Analyses of Variance (ANOVAs) indicated a significant effect of diagnosis on each domain of everyday EF (*p* < .05) and depression symptoms (*p* = .007) (see Table S2). Pairwise comparisons indicated that the ADHD and ADHD+DCD groups had significantly greater EF difficulties than the DCD group on all domains of everyday EF (*p* < .05) except for organisation of materials. In addition, the ADHD+DCD group had significantly higher levels of depression symptoms than the ADHD and DCD groups (*p* < .05) (see Table S3).

**Table S2**

*Means, Standard Deviations, and ANOVAs of Group Differences in EF (BRIEF-A) and Depression Symptoms (MFQ)*

| Measure | | ADHD  group | | DCD  group | | ADHD+DCD group | | *F* | *p* | η^2^ |
| --- | --- | --- | --- | --- | --- | --- | --- | --- | --- | --- |
|  |  | *M* | *SD* | *M* | *SD* | *M* | *SD* |  |  |  |
| BRIEF-A |  |  |  |  |  |  |  |  |  |  |
|  | GEC | 81.9 | 8.76 | 71.2 | 10.8 | 82.2 | 8.46 | 21.58 | <.001 | .24 |
|  | BRI | 77.5 | 9.89 | 66.3 | 11.6 | 77.5 | 10.3 | 18.77 | <.001 | .22 |
|  | MI | 81.7 | 10.5 | 72.1 | 10.5 | 81.7 | 8.77 | 15.29 | <.001 | .18 |
|  | Inhibit | 74.1 | 8.78 | 61.5 | 11.4 | 73.7 | 8.58 | 26.57 | <.001 | .28 |
|  | Shift | 73.6 | 11.6 | 67.2 | 11.5 | 76.4 | 12.7 | 7.15 | .001 | .1 |
|  | EC | 72.5 | 11.9 | 64.9 | 11.3 | 71.7 | 10.2 | 7.22 | .001 | .1 |
|  | S-M | 69.6 | 11.1 | 59.3 | 12.4 | 68.7 | 14.6 | 11.49 | <.001 | .14 |
|  | Initiate | 75.3 | 10.3 | 65.8 | 11.1 | 75.5 | 9.7 | 14.07 | <.001 | .17 |
|  | WM | 83.7 | 9.02 | 77.7 | 10.9 | 84.8 | 9.94 | 6.93 | .001 | .09 |
|  | P/O | 78.6 | 10.2 | 68.4 | 10.9 | 78.2 | 10.1 | 15.74 | <.001 | .19 |
|  | TM | 78.6 | 10.4 | 68.5 | 11.2 | 77 | 9.74 | 14.2 | <.001 | .17 |
|  | OM | 69.4 | 12.4 | 65 | 11.1 | 71 | 9.86 | 3.31 | .04 | .05 |
| MFQ |  | 28.3 | 13.6 | 26.4 | 13.3 | 37.1 | 16.6 | 5.09 | .007 | .07 |

*Note.* GEC = Global Executive Composite; BRI = Behavioural Regulation Index; MI = Metacognition Index; EC = Emotional Control; S-M = Self-Monitor; WM = Working Memory; P/O = Plan/Organize; TM = Task Monitor; OM = Organization of Materials.

**Table S3**

*Pairwise comparisons using Tukey HSD on group differences in BRIEF-A and MFQ scores.*

| Measure | Group | Mean difference | *p* |
| --- | --- | --- | --- |
| BRIEF-A |  |  |  |
| GEC | ADHD - DCD  ADHD+DCD - DCD  ADHD+DCD - ADHD | 10.79  11.02  0.23 | <.001  <.001  .99 |
| BRI | ADHD - DCD  ADHD+DCD - DCD  ADHD+DCD - ADHD | 11.24  11.18  -.06 | <.001  <.001  .99 |
| MI | ADHD - DCD  ADHD+DCD - DCD  ADHD+DCD - ADHD | 9.57  9.6  .04 | <.001  <.001  .99 |
| Inhibit | ADHD - DCD  ADHD+DCD - DCD  ADHD+DCD - ADHD | 12.6  12.22  -.38 | <.001  <.001  .99 |
| Shift | ADHD - DCD  ADHD+DCD - DCD  ADHD+DCD - ADHD | 6.38  9.24  2.85 | .01  .004  .6 |
| Emotional Control | ADHD - DCD  ADHD+DCD - DCD  ADHD+DCD - ADHD | 7.54  6.76  -.78 | .002  .04  .96 |
| Self-Monitor | ADHD - DCD  ADHD+DCD - DCD  ADHD+DCD - ADHD | 10.29  9.36  -.92 | <.001  .006  .95 |
| Initiate | ADHD - DCD  ADHD+DCD - DCD  ADHD+DCD - ADHD | 9.51  9.69  .18 | <.001  .001  .99 |
| Working Memory | ADHD - DCD  ADHD+DCD - DCD  ADHD+DCD - ADHD | 5.97  7.11  1.15 | .001  .01  .89 |
| Plan/Organize | ADHD - DCD  ADHD+DCD - DCD  ADHD+DCD - ADHD | 10.18  9.73  -.45 | <.001  .001  .98 |
| Task Monitor | ADHD - DCD  ADHD+DCD - DCD  ADHD+DCD - ADHD | 10.1  8.5  -1.6 | <.001  .004  .82 |
| Organization of Materials | ADHD - DCD  ADHD+DCD - DCD  ADHD+DCD - ADHD | 4.39  5.91  1.52 | .1  .09  .86 |
| MFQ | ADHD - DCD  ADHD+DCD - DCD  ADHD+DCD - ADHD | 1.9  10.75  8.85 | .75  .005  .04 |

*Note*. EC = Emotional Control; S-M = Self-Monitor; WM = Working Memory; P/O = Plan/Organize; TM = Task Monitor; OM = Organization of Materials.

**EF and Depression Symptoms as Predicted by ADHD and DCD Symptoms**

The multiple linear regression models testing the predictive effects of ADHD and DCD symptoms on each domain of EF and depression symptoms, without covariates, were all significant (*p* < .05). ADHD symptoms was entered first into models, followed by DCD symptoms. Where DCD symptoms were a significant predictor, incremental F-ratio tests confirmed they explained a significant amount of variance (*p* < .05) over and above ADHD symptoms. Both ADHD and DCD symptoms were significant predictors of EF and depression symptoms in the full sample (p < .05). The regressions run within each diagnostic group revealed that ADHD symptoms was a significant predictor of EF in the DCD and ADHD+DCD groups (*p* < .05), while DCD symptoms were significant predictors of EF and depression symptoms in the ADHD group (*p* < .01; see Table S4).

**Table S4**

*Prediction of Overall EF (GEC) and Depression Symptoms (MFQ) from ADHD Symptoms (ASRS) and DCD Symptoms (ADC)*

| Predictor | GEC | | | MFQ | | |
| --- | --- | --- | --- | --- | --- | --- |
|  | Estimate (β) | 95% CI | *p* | Estimate (β) | 95% CI | *p* |
|  | Full Sample | | | | | |
| ASRS | .71 | [.56, .8] | <.001 | .22 | [.06, .49] | .01 |
| ADC | .14 | [.01, .23] | .03 | .32 | [.12, .52] | .002 |
|  | ADHD group | | | | | |
| ASRS | .24 | [-.03, .52] | .07 | -.13 | [-.73, .33] | .45 |
| ADC | .51 | [.16, .52] | <.001 | .48 | [.15, .85] | .006 |
|  | DCD group | | | | | |
| ASRS | .73 | [.51, .89] | <.001 | .37 | [-.03, .92] | .06 |
| ADC | .05 | [-.14, .24] | .6 | .13 | [-.17, .46] | .36 |
|  | ADHD+DCD group | | | | | |
| ASRS | .51 | [.1, .82] | .01 | .3 | [-.41, 1.48] | .27 |
| ADC | .37 | [-.01, .49] | .06 | .12 | [-.42, .73] | .61 |

*Note.* β = Standardized coefficient.

Multiple linear regression model exploration of the individual domains of EF revealed that ADHD symptoms was a significant predictor of each domain of EF (*p* < .01), and DCD symptoms for metacognition, shifting, working memory, task monitoring, and organisation of materials (*p* < .05; see Table S5).

**Table S5**

*Prediction of EF (BRIEF-A) from ADHD Symptoms (ASRS) and DCD Symptoms (ADC)*

| Predictor | Estimate (β), 95% CI, *p* | | | | | | | | | | |
| --- | --- | --- | --- | --- | --- | --- | --- | --- | --- | --- | --- |
|  | BRI | MI | Inhibit | Shift | EC | S-M | Initiate | WM | P/O | TM | OM |
| ASRS | β = .68, [.57, .86],  *p* <.001 | β = .63, [.45, .77],  *p* <.001 | β = .83, [.73, .97],  *p* <.001 | β = .31, [.16, .5],  *p* <.001 | β = .49,  [.32, .67],  *p* <.001 | β = .57, [.48, .83],  *p* <.001 | β = .59, [.44, .75],  *p* <.001 | β = .59, [.41, .67],  *p* <.001 | β = .55, [.41, .71],  *p* <.001 | β = .59,  [.46, .74],  *p* <.001 | β = .28,  [.1, .46],  *p* = .002 |
| ADC | β = .05,  [-.08, .18],  *p* =.47 | β = .16, [.02, .26],  *p* = .02 | β = -.11,  [-.21, .01],  *p* = .09 | β = .32,  [.15, .47],  *p* <.001 | β = -.01,  [-.17, .15],  *p* = .88 | β = .06,  [-.1,.22]  *p* = .46 | β = .03,  [-.11, .17],  *p* = .7 | β = .17, [.02, .26],  *p* = .02 | β = .15, [-.001, .27],  *p* = .05 | β = .16,  [.02, .28],  *p* = .03 | β = .21,  [.03, .35],  *p* = .02 |

*Note.* β = Standardized coefficient. GEC = Global Executive Composite; BRI = Behavioural Regulation Index; MI = Metacognition Index; EC = Emotional Control; S-M = Self-Monitor; WM = Working Memory; P/O = Plan/Organize; TM = Task Monitor; OM = Organization of Materials.

## Depression Symptoms as Predicted by EF

The simple linear regression models examining each domain of everyday EF as a predictor of depression symptoms, without covariates, were all significant (*p* < .001) except for the model with organisation of materials as the predictor (*p* > .05). The regressions suggested that overall EF was a significant predictor of depression symptoms across the full sample and each diagnostic group (*p* < .05). The remaining domains of everyday EF significantly predicted depression symptoms in the full sample (*p* < .001), except for organisation of materials (*p* > .05; see Table S6).

**Table S6**

*Prediction of Depression Symptoms (MFQ) from EF (BRIEF-A)*

| Predictor | MFQ | | |
| --- | --- | --- | --- |
|  | Estimate (β) | 95% CI | *p* |
|  | Full Sample | | |
| GEC | .48 | [.44, .82] | <.001 |
| BRI | .51 | [.44, .78] | <.001 |
| MI | .38 | [.29, .69] | <.001 |
| Inhibit | .38 | [.27, .65] | <.001 |
| Shift | .41 | [.3, .66] | <.001 |
| EC | .5 | [.43, .79] | <.001 |
| S-M | .34 | [.2, .55] | <.001 |
| Initiate  WM | .46  .3 | [.39, .76]  [.19, .63] | <.001  <.001 |
| P/O | .39 | [.29, .67] | <.001 |
| TM | .31 | [.19, .58] | <.001 |
| OM | .12 | [-.06, .36] | .15 |
| GEC | 0.53 | ADHD group  [.45, 1.21] | <.001 |
| GEC | .46 | DCD group  [.29, .84] | <.001 |
| GEC | .42 | ADHD+DCD group  [.18, 1.61] | .01 |

*Note.* β = Standardized coefficient. GEC = Global Executive Composite; BRI = Behavioural Regulation Index; MI = Metacognition Index; EC = Emotional Control; S-M = Self-Monitor; WM = Working Memory; P/O = Plan/Organize; TM = Task Monitor; OM = Organization of Materials.

## Mediation Effects of EF in the Relation between ADHD/DCD and Depression Symptoms

Following up on the significant findings from the results above, simple mediation analyses were run to test the indirect relationships between (1) ADHD and depression symptoms via each domain of EF, excluding organisation of materials; and (2) DCD and depression symptoms via each of the following: overall EF, metacognition, shifting, working memory, and task monitoring.

As shown in Table S7, overall EF, behavioural regulation, inhibition, shifting, self-monitoring, emotional control, initiation, and planning/organising respectively acted as significant mediators of associations between ADHD and depression symptoms, however metacognition, working memory, and task monitoring did not show mediating effects. Overall EF, behavioural regulation, inhibition, emotional control, initiation, and planning/organising displayed full mediating effects, while shifting and self-monitoring showed partial mediating effects. Additionally, overall EF, metacognition, and shifting respectively showed significant partial mediating effects between DCD and depression symptoms. Working memory and task monitoring did not present mediating effects.

**Table S7**

*Results of the Simple Mediation Models (ADHD/DCD Symptoms → Everyday EF → Depression Symptoms)*

| Mediation model | Estimate (β), 95% CI | | | | |
| --- | --- | --- | --- | --- | --- |
|  | Effect of IV on M | Effect of M on DV | Total Effect | Direct Effect | Indirect Effect |
|  | ADHD symptoms → Everyday EF → Depression symptoms | | | | |
| ASRS → GEC → MFQ | β = .77, [.62, .85] * | β = .52, [.37, .98] * | β = .36, [.23, .7] * | β = -.04, [-.34, .27] | β = .4, [.27, .74] * |
| ASRS → BRI→ MFQ | β = .71, [.6, .86] * | β = .52, [.35, .9] * | β = .36 [.21, .7] * | β = -.01, [-.33, .33] | β = .37, [.24, .71] * |
| ASRS → MI → MFQ | β = .7, [.55, .81] * | β = .25, [-.01, .62] | β = .36, [.2, .68] * | β = .18, [-.06, .55] | β = .18, [-.01, .42] |
| ASRS → Inhibit → MFQ | β = .78, [.69, .9] * | β = .25, [.02, .62] * | β = .36, [.2, .69] * | β = .16, [-.12, .52] | β = .2, [.02, .51] * |
| ASRS → Shift → MFQ | β = .47, [.33, .64] * | β = .31, [.16, .57] * | β = .36, [.21, .69] * | β = .21, [.03, .53] * | β = .14, [.08, .31] * |
| ASRS → EC → MFQ | β = .48, [.33, .64] * | β = .43, [.3, .74] * | β = .36, [.22, .68] * | β = .15, [-.05, .45] | β = .21, [.13, .39] * |
| ASRS → S-M → MFQ | β = .6, [.55, .82] * | β = .2, [.01, .44] * | β = .36, [.21, .69] * | β = .24, [.01, .58] * | β = .12, [.01, .32] * |
| ASRS → Initiate→ MFQ | β = .61, [.47, .75] * | β = .39, [.27, .7] * | β = .36, [.19, .69] * | β = .12, [-.11, .45] | β = .24, [.16, .44] * |
| ASRS → WM → MFQ | β = .67, [.47, .73] * | β = .1, [-.16, .45] | β = .36, [.22, .69] * | β = .29, [.09, .66] * | β = .07, [-.1, .25] |
| ASRS → P/O → MFQ | β = .63, [.51, .75] * | β = .27, [.08, .59] * | β = .36, [.21, .7] * | β = .19, [-.03, .54] | β = .17, [.05, .39] * |
| ASRS → TM → MFQ | β = .67, [.55, .79] * | β = .13, [-.1, .41] | β = .36, [.2, .69] * | β = .27, [.06, .62] * | β = .09, [-.07, .28] |
|  | DCD symptoms → Everyday EF → Depression symptoms | | | | |
| ADC → GEC → MFQ | β = .48, [.3, .54] * | β = .39, [.25, .75] * | β = .39, [.26, .62] * | β = .21, [.04, .42] * | β = .19, [.1, .33] * |
| ADC → MI → MFQ | β = .46, [.29, .52] * | β = .25, [.07, .56] * | β = .39, [.26, .6] * | β = .27, [.12, .48] * | β = .12, [.03, .24] * |
| ADC → Shift → MFQ | β = .47, [.3, .58] * | β = .29, [.13, .56] * | β = .39, [.26, .63] * | β = .26, [.07, .49] * | β = .14, [.05, .28] * |
| ADC → WM → MFQ | β = .46, [.26, .49] * | β = .15, [-.04, .49] | β = .39, [.27, .61] * | β = .32, [.17, .55] * | β = .07, [-.02, .19] |
| ADC → TM → MFQ | β = .45, [.3, .53] * | β = .17, [-.01, .45] | β = .39, [.28, .62] * | β = .31, [.17, .53] * | β = .08, [-.003, .19] |

*Note.* β = Standardized coefficient. IV = Independent variable. M = Mediator. DV = Dependent variable. GEC = Global Executive Composite; BRI = Behavioural Regulation Index; MI = Metacognition Index; EC = Emotional Control; S-M = Self-Monitor; WM = Working Memory; TM = Task Monitor.

*: Significant confidence interval
